# Treatment of Fracture of the Jaw

**Published:** 1884-02

**Authors:** Thomas Brian Gunning

**Affiliations:** New York.


ARTICLE II.

### TREATMENT OF FRACTURE OF THE JAW, WITH CRITICAL REMARKS, AS SENT TO PROFESSOR D. HAYES AGNEW, M. D.

(Continued.)

---

BY THOMAS BRIAN GUNNING, D. D. S., NEW YORK.

---

In addition to all this your own City, Philadelphia, has in one of its prominent men a proof of the superiority of my treatment by splint over that by bandage as used by Professor Buckingham of the Medical School of Harvard University. The fracture was received in the terrible stage accident in the White Mountains in 1873, and the jaw when the patient was brought to me, was so deformed that I had to break it apart. The splint was shown in the Centennial Exhibition of 1876 and the case is fully reported in the *Independent Practitioner*, Vol. 1, p. 526.

I have said that you leave out my splint 4; but worse still you place next after my Fig. 3 a splint which you show as Kingsley's preceded by a description which ends as follows: "When applied, the teeth occupy the cavities in the splint, the latter being kept in position by a strip of roller passing beneath the chin from one arm of the apparatus to the other."

Now this method, the splint and the wings (arms) were devised by me.

The splint with its wings was shown on the patient to the New York Academy of Medicine, October 21st, 1863.

It was for showing this apparatus that the Academy passed the resolution thanking me and requesting me to report further; and in response to which I read the paper, June 1st, 1864, which explained my four splints. (See *Bulletin* Vol. 2, pp. 153, 168 and 307.

Kingsley does not use the splint nor the roller as you say, but applies a sub-mental splint or compress which is by means of some apparatus or band kept in connection with the interdental splint, the broken jaw being between them, and he maintains that this is necessary. But for fracture at the angle of the jaw or in the parts above, he says that an interdental splint is useless and that a bandage is indicated, that is a bandage around the jaw and head. Thus Kingsley uses appliances external to the mouth in all cases although my interdental splint, which by enclosing all the lower back teeth, holds in the angles of the jaw, has been in use since February 12, 1864, his treatment of "Fracture of the Jaw" is no improvement upon that of twenty-five years ago. In fact he advises for fracture in front of the jaw, that which is inferior to Hayward's plan of 1858, in which a metal cap was fitted to several teeth on each side of the fracture and from the upper surface of the cap a stout wire went out of each corner of the mouth to a gutta-percha splint under the chin, and from beneath this a four-tailed bandage passed behind and over the head, thus the lower tails passed outside the angles which could therefore be held in with pads. Whereas, Kingsley says: "If the fracture is in front, the splint need not cover all the back teeth; but if it be at the side, it is better to cover all the teeth of that side. It is also better to set the ends of the upper and lower jaws in an articulator, and thus make prints of the upper teeth in the wax, to be retained in the "splint." But with the splint cut off so as not to enclose the back teeth, the angles will be forced out by the muscular traction on the inside of the chin, for the outside wings can not be used beyond the ends of the splint as they would lift it up from the front teeth. In fact the muscular traction on the inside of the chin might alone wring the fractured ends out of the splint by forcing the latter up the outer surfaces of the canine or bicuspid teeth.

Again if the splint only covered the teeth on the fractured side, then those of the other would have no bearing and no eating could be done except on the splint over the fracture, and if the patient should happen to use the uncovered teeth on a large morsel it might force the fractured ends apart whether they were in the side or in the front of the jaw. Dr. Kingsley's examples of the application of this splint to double or triple fractures are quite as bad as his advice in regard to single fractures. All he shows of importance was first devised, used and published by others before him; it is as told by him of little service to the reader because of its intermixture with statements which are not in accord with the facts of history nor of science.

Yet your text is such as leads the reader to adopt his treatment and reject my methods this not because of any want of clearness in my description for you could have quoted from my paper in the *New York Medical Journal*, Vol. 3, p. 434, "When a well adapted splint is on the teeth and gum, the other parts around the bone are, to a great extent a counter-support to the splint * * * * Meanwhile the motions of the jaw are in most cases unrestricted, and the cheeks and lips always left free." I bid 442 " Fig. 1 is the representative splint for treatment of cases in the first-class or those in which the jaw is left free. Fig. 2 for the second class, or those in which the jaw is held still."

Yet with this plainly stated you class these splints together and say, " This splint when placed in position, forms a cap, and is kept in place by securing the jaws together with a bandage, or by means of screws passed between the teeth." Your text on page 847 confirms this as follows : " The splint of Dr. Bean resembles closely that of ' Dr. Gunning ' * * * * * ' The splint when applied is kept in position by straps which pass over and around the head, and also behind the neck.' Your text again misleads the student, for you admit that Beans' Splint ' is fitted to the teeth of both the upper and lower maxilla,' in which cases my splint is screwed to the teeth and while I use nothing outside, the mouth, you lead the reader to suppose that I use a bandage around the jaw and head and you do this although my treatment of fracture, with the splint illustrated by Fig. 2, (your Fig. 643) is related in the *New York Medical Journal*, Vol. 4, p. 16. In case 5 it is distinctly shown that the surgeon in Bellevue Hospital who had charge of the woman tried to hold the jaw up in the splint with Hamilton's bandage but on the third day he requested me to screw the splint fast to the teeth, as the bandage was painful and useless. The splint was screwed to the teeth and the jaw united in forty days.

In January, 1861, Dr. Benjamin Franklin Bache, United States Navy, advised that I should be asked to treat a Spanish seaman, whose fractured jaw was found to be incurable at the Naval Hospital, New York. Howard Hayward's method of treating these injuries was then the most advanced and although it was very imperfect did much to prepare the minds of surgeons to accept the co-operation of dentists, at least in Great Britian. After study of the literature of the subject say as given for twenty-three centuries back and then making careful allowance for the muscular action involved, I devised the splints and methods of applying them, which were fully published ; and the correct action of the muscles which control the jaw was shown to guide the surgeon. This was necessary as I demonstrated that the *external pterygoid* muscles depressed the jaw and opened the mouth, instead of the *digastrics* as maintained by Hunter. I also explained that the lower jaw is the lever by which the head is held forward so that when the jaw is broken, it requires firmer control than can be given by appliances which rest upon the soft parts external to the mouth. In the years which have since passed, my experience has suggested nothing which I think necessary to perfect my treatment of fractures of the maxillæ.

You however do not show my splints and methods *fairly* in order that your readers may contrast my plans with others and judge of their relative merits as the student is led to believe by your preface.

I trust that you have been yourself imposed upon, and that you will now and in the future as far as possible correct the wrong which has at present the sanction of your name.

I am, Sir,

Yours Respectfully,

THOMAS BRIAN GUNNING.

---

(Copy.)

PHILADELPHIA,

1611 CHESTNUT STREET,

*December 1st, 1883.*

DR. THOMAS BRIAN GUNNING,

*Dear Doctor :—*

I am indebted to you for an accurate description of your interdental splints for fracture of the jaw, and shall with great pleasure make the correction in the next edition of my book should it reach a second edition.

Very Truly Yours, &c.,

D. HAYES AGNEW.

